# A Clinical and Therapeutical Treatise on Hysteria

**Published:** 1861-03

**Authors:** 


### NORTH AMERICAN

### MEDICO-CHIRURGICAL REVIEW.

**MARCH, 1861.**

### Analytical and Critical Reviews.

Art. I.—*Traité Clinique et Therapeutique de l'Hysterie.* Par le Docteur P. Briquet, Médecin à l'Hôpital de la Charité, Agregé Honoraire de la Faculté de Médecine de Paris, etc. Paris: Bailliere et Fils, 1859. 8vo., pp. 724.

*A Clinical and Therapeutical Treatise on Hysteria.* By Doctor Briquet, Physician to the Charity Hospital, Honorary Associate of the Faculty of Medicine of Paris, etc. Paris. 8vo., pp. 724.

A physician, who in ten years of hospital practice has made careful observations on four hundred and thirty patients afflicted with hysteria, may well claim the attention of his medical brethren when he discourses on this disease. M. Briquet has thus enjoyed rare opportunities for becoming the historian of hysteria; and the volume, whose title we have just given, must be received as evidence of the ability and critical impartiality with which he has fulfilled his self-imposed duty. It will be ours to follow in the course which he has so successfully pursued, and to repeat some of the leading facts with the accompanying numerals, with which nearly every chapter of the work is interspersed. Speculations and conjectures would be entirely misplaced on the present occasion, nor can we even indulge our readers with the retrospective lore introduced by M. Briquet, as when he gives an account of the opinions entertained by medical writers, from the time of Hippocrates down to the present day, on the causes and essential character of hysteria. It is sufficient for us to say, that all the ancient and a large majority of modern writers on the subject, taught that hysteria was a uterine affection, or rather a disease having its origin in the uterus, however numerous and diversified might be the subsequent and associated disturbance in other organs. The disorder of this organ, causing strong sexual and unsatisfied venereal desire, and the subsequent train of nervous symptoms, was, according to the pathology so long dominant, to be cured by sexual intercourse. To this view stand opposed the facts adduced in the volume before us, viz., that hysteria is met with in the other sex, and in young females before the age of puberty, and before the uterus takes on any specific action, or is either the recipient or dispenser of impressions of any kind. Nor is immunity given by the cessation of the uterine functions in a later period of life. It is known, moreover, that the disease shows itself in subjects in whom there is no uterus.

M. Briquet, under the head of "Definition," declares his belief that hysteria is a neurosis of the encephalon, the obvious phenomena of which consist chiefly in a disorder of the vital acts which serve to manifest the affective sentiments and the passions. The changes peculiar to each affective sentiment and each passion in the human species cover the field of hysteria. Every hysteric phenomenon has its true type in the different vital acts by which the affective sentiments and the passions are manifested externally. All the hysteric disorders which appear to be so anomalous, and which for a long time puzzled physicians, are nothing more than the pure and simple repetition of these acts, but augmented, weakened, or perverted. We may take any symptom whatever of hysteria, and we shall always find its model in one of the acts which constitute passionate manifestations. The author, in further illustration of his meaning, instances the case of an excitable female who has just been subjected to a sudden and strong emotion. She experiences, at the very moment, a constriction of the epigastrium, and a feeling of oppression; her heart beats strongly; something comes up in her throat and chokes her; her limbs are the seat of a wearied feeling and refuse to perform their office, so that she is on the point of falling down; or, again, she experiences a restlessness, a call for movement, which induces active contraction of the muscles. Here we have the exact counterpart of an ordinary hysteric attack with spasms. The frequent repetition of these manifestations ends in dynamic or material lesions in the organs primarily affected, and thus adds new complications to the already bewildering scenes exhibited in hysteria.

These views are, M. Briquet remarks, very different from the crude theories which can only suppose in hysteria ungratified appetites, or the inflammation, suppuration, or cancer of the genital organs, but they accord with the more philosophic opinions of Raulin and Sydenham, and many other modern writers. The testimony given by the author might have been extended had he borrowed more freely from English medical literature. In the latter, hysteria is attributed to a morbid condition of the spinal marrow, but the authors of this opinion still leave unexplained the remoter seat of the irritation, which calls into morbid action the organs under spinal influence. This difficulty, it must be confessed, stands in the way of our entire adoption of the cerebral nature of the disease, as advocated by M. Briquet.

"Hysteria acknowledges for its chief symptoms extreme sensibility of the nervous system, various hyperæsthesiæ, among which are most prominent, pains in the epigastric region, in the left side of the thorax, along the left vertebral fossa, anæsthesia principally affecting the skin, the muscles, and the organs of the senses; spasms, particularly at the epigastrium, the sensation as of a globe mounting from the œsophagus to the throat, and of choking; finally, convulsions, which are commonly accompanied by loss of consciousness, and which end in tears and sobbing—symptoms, all of which are under the influence of the moral affections."

This may serve as a description of the essential features of hysteria, but conveys a very imperfect idea of the variety of symptoms expressive of organic disease, which follow in its train, or are associated with it. The author quotes Riverius, who speaks of it as an Iliad of diseases rather than a single disease, and Sydenham, who says that the changes of form of Proteus and of the hues of the cameleon, are not more various than the different aspects under which hysteria presents itself to our notice. Hoffmann defines it thus, "*Morbus ille, aut potius morborum cohors*," etc.

In entering on the "etiology" of hysteria, to which the *first part* of the volume is given, M. Briquet adopts the received division of causes, viz., the predisposing and the determining or exciting, although he admits that these two series of causes may run into each other, and sometimes even change their relative signification, so that a predisposing becomes by its duration an exciting cause. He studies the predisposing causes of the disease under the heads of—1, sex; 2, age; 3, the state of health of the parents; 4, the physical and moral constitution; 5, climate; 6, the social position; 7, the place in which the person has been educated; 8, the mode of education; 9, the kind of alimentation; 10, the profession or calling; 11, the passions; 12, continence; 13, the state of menstruation; 14, antecedent diseases, and especially those of the genital organs; 15, and finally, the state of health of women before the date at which the exciting causes have begun to act.

First, then, as regards "the influence of sex" in predisposing to hysteria. For those writers who look on the uterus as the exclusive seat of hysteria, or, at the least, the organic centre whence radiates all the irritations that make up the sensible evidences of the disease, the question of sex would be a useless one, since the disease, according to their theory, can only appear in females. But while the great majority of hysteric subjects are found among the latter, it is not the less clear that persons of the male sex furnish undoubted examples of its occurrence among them.

Louyer Villermay himself, while asserting that man cannot become hysteric, because, as this writer says, he has no uterus, admits, nevertheless, that he may be subjected to very peculiar nervous affections, and even convulsive attacks, analogous to those which constitute hysteria. Cases are cited from medical journals, some of them in detail, of hysteria in men. To the same purport reference is made to the inaugural theses of MM. Bonneau, Legoarent, and Desterne. M. Briquet has seen a certain number of cases of hysteria in men, the records of which he gives on the present occasion. We regret, for the sake of our readers, that we cannot reproduce them here, but room is wanting for the purpose, and we must pass on. Of the seven cases recorded in detail, five were simple hysteria, unconnected with any other disease, and two were complicated; one of these with general paralysis and rigidity of the muscles, the other with progressive paralysis, which terminated fatally. Two cases of hysteria in men have come under our own observation. We were called up in the night to visit one of them, who was suffering from a clearly marked attack, in which the *globus hystericus* was a prominent symptom. Both of these persons were excitable and nervous subjects, though differing not a little from each other in their intellectual and moral qualities. The attacks of the one could be traced to mental emotions, those of the other to indigestion arising from gluttonous indulgences and deficient exercise.

It has been alleged by those who advocate the uterine pathology of hysteria, that the cases in which the disease has been witnessed in man owe their origin to excitement of his genital organs, and non-gratification of the venereal appetite; but, as is shown by the author, a rigid analysis of the cases recorded by different writers, including those which came under his own observation, fail to bear out this view. The two hysterical patients above referred to, coming under our own care, were both of them married men.

The fact must not, however, be for a moment lost sight of, viz., that hysteria is the apanage of women, among whom a thousand cases have been collected for fifty in men. In females generally a fourth of the number are subject to hysteria, and more than half of them are either hysterical or very excitable. What is the reason of this sad preference given to them? "Evidently it can be attributed only to three orders of causes: to the particular constitution of the genital organs of the female; to the special nature of her organization in general; and to that of her nervous system in particular." M. Briquet proceeds to investigate the actual and relative share of these three conditions for the supervention of hysteria. Regarding the first, we have the early dictum of Hippocrates: *Mulier propter uterum condita est;* and that of Van Helmont: *propter solum uterum, mulier est id quod est.* Equally strong is the creed of M. Dubois d'Amiens, that, in fact, the uterus makes the woman, during the mean period of her life. Unfortunately for this opinion, replies our author, a certain number of instances are on record of women in whom the uterus was entirely wanting, but who exhibited, nevertheless, the characteristics of the sex, and fulfilled entirely their connubial obligations as *femmes couvertes*.

The author gives a case coming under his own observation of a woman twenty-four years of age, who died in the hospital from typhoid fever, and in whom, as was ascertained by an autopsy, the uterus was entirely wanting; the Fallopian tubes were represented by two fibrous cords, each terminating at the rudiments of ovaries, which were replaced by a grayish tissue. The vagina was only six to seven centimetres (about two and one-half inches) in length, and terminated in a solid tissue; it formed in fact a true *cul-de-sac*. The pelvis was normal and full, as in women generally; the pubis was furnished with hair resembling that found in the adult woman; the greater and lesser labiæ, as well as the corpora cavernosa and the clitoris, were moderately developed. This young woman had none of the attributes of the other sex; her face and features were mild and delicate, and there was no beard on the upper lip; her voice was feminine. The limbs were round and full, and without muscular prominences; the chest was narrow and the haunches were very full. On inquiry as to her character and habits, it was ascertained that they were those of a woman, and moreover that she had a lover, to whom, from complaisance, as she said, she was in the habit of allowing everything.

That the uterus does not make the woman is further proved by reference to her early or infantile life, when the uterus and the entire genital apparatus are in a rudimentary state, and perform no specific function, and yet even thus early both the physical and moral peculiarities of the sex are evinced, and stand in marked contrast with those of boys of the same age.

He next proceeds to consider the influence of the nervous system in the production of the disease, and remarks truly, what we are fain to admit as an ultimate fact without our being able to explain it by anatomy, that woman has greater sensibility, both physical and moral, than man, whether we look at her tactile sensibility, her delicacy of taste, and susceptibility to any external impression, even to that of strong odors. So sensitively disposed is the sex that it is rare to see a woman who, under any strong emotion, does not suffer in the manner described in a preceding paragraph.

*Influence of age on hysteria.*—This, as M. Briquet justly remarks, is a question not merely of facts, but it is an important element in the solution of the problem of the nature of this disease.

Looking for facts, we find in 351 cases collected by M. Landouzy (*Traité de l'Hysterie*) from the writers who preceded him, the following proportions: Hysteria showed itself in 48 persons, between the ages of 10 and 15 years; in 105, between 15 and 20; in 80, between 20 and 25; in 40, between 25 and 30; in 38, between 30 and 35; in 15, from 35 to 40; in 7, from 40 to 45; in 8, from 45 to 50; in 4, from 50 to 55; in 4, from 55 to 60; in 1, from 60 to 65; and in 1, from 80 to 85. M. Landouzy omitted to introduce into his tabular statement four cases of hysteria which occurred before ten years of age, probably under an impression that, with the prevalent doctrine of the etiology of the disease, they would not be admitted to be genuine cases. Georget, at the Salpetrière, saw a case of hysteria in a female nine years of age; and M. Beau, in the same establishment, records, in 19 cases of hysteria, 6 which occurred between 10 and fifteen years of age. M. Briquet exhibits a tabular statement of the proportionate number of cases of hysteria during the period of infancy, as he terms it, and between this and puberty, in a grand total of 87 cases. In the first or early infantile period, ending at five years of age, there were 31 cases, of which 14 were accompanied by convulsive attacks, and 17 by states of suffering which must be regarded as special to hysteria. In the second of the two above-mentioned periods, 3 had regular attacks (convulsions, etc.) at 5 years of age; 6, between 6 and 7 years, also with convulsions; 11, between 7 and 8 years; 6, between 8 and 9 years; 9, between 9 and 10 years; 4, between 10 and 11 years; and 17, between 11 and 12 years. It appears, therefore, from this numerical statement, that hysteria manifested itself in eighty-seven cases in a period ranging from infancy to puberty, and, consequently, that one-fifth of all the cases noted by the author were anterior to the age of puberty. The history of some of these is furnished in the volume before us, and we must again express our regret at not being able to introduce them into our pages.

The predisposition to hysteria, so far as it depends on the nervous system, and the functional movements occurring in the latter, is shown, manifestly, to bear a closer relation to age than to any other etiological or modifying circumstance. Rank in life does not alter the relative number of cases of predisposition to the disease depending on age. They were the same in the more elevated classes as reported by M. Landouzy, as those of the poorer class, inmates of the hospital, recorded by M. Briquet. A fifth of the number of hysterical patients, we repeat, had become so before the age of puberty. The period of the maximum number of these persons was between twelve and twenty years of age—a period in which two-fifths of the subjects of hysteria are met with. The predisposition diminishes from 20 to 25 years. It is very slight from 25 to 40 years; and hardly exists at all between 50 and 60 years. It is obvious that the predisposition to hysteria before puberty cannot have any connection with the state of the genital organs; nor can we believe in any influences of this nature being exerted at a later period, as between 19 and 25 years of age, when there is decreased predisposition to the disease, notwithstanding the active state of these organs during this time. If, on the other hand, we look to the susceptibilities growing out of the activity of the encephalic organs, we shall find adequate explanation of the differences in predisposition during these two periods.

The *hereditary* predisposition to hysteria would seem to be proved on good evidence. M. Briquet cites a number of authors to this effect, and adds his own confirmatory statements, based on a large number of cases. In summing up, M. Briquet tells us, that the children of hysterical parents are twelve times more subject to hysteria than are equal numbers of those whose parents have not been afflicted with hysteria. Omitting the statistical returns introduced by the author, we give his conclusion, viz., that half of the number of hysterical mothers give birth to children who in their turn become hysterical.

An inquiry into the "*physical constitution and the moral nature*," as influencing causes of hysteria, leads M. Briquet to infer that the predisposition to the disease is chiefly due to moral influences; and more particularly to those of a painful nature. The inferences laid down by the author are reached after a citation of facts and reasoning for which we have not room on this occasion.

Touching the influence of *climate* in the production of hysteria, we are told that this disease is found in all climates. If warm climates seem to give rise to hysteria, the cause must be sought for in the frequent indulgence and liveliness of the passions, originating in the manners of the people who inhabit them.

The conclusions drawn from observation of the effects of *social position* as relates to hysteria are such as would occur to most inquirers, viz., that although all women, rich and poor, may become hysterical, yet will the frequency be proportionate to the ills endured, and that as the poor suffer most they will count the larger number of victims to the disease.

A contrast has sometimes been attempted to be drawn between females born and brought up in the *city*, and those who were natives of and lived in the *country*, in favor of the latter. The difference is not, however, M. Briquet assures us, so great as is generally supposed. Hysteria occurs at a more advanced age in the country than in a city, but convulsions seize those living in the former less readily than those in the latter. In great centres of population, such as very large cities, hysteria is unquestionably very common, as we may readily believe from a union of all the causes which enfeeble the organism and give an undue predominance to the nervous system.

"*Education*" influences the state of susceptibility to hysteria, the readiness or the occurrence of which will be increased by idleness, frivolous occupations, crowded company, the ball-room and the theatre, reading of novels and exaggerated pictures of life. But the opposite, or harshness and undue restraint and maltreatment in early life, will produce equal evils, by deteriorating the health and predisposing to, and at times directly causing hysteria.

*The alimentary regimen*, in as far as it is operative in disposing to hysteria, does so, as M. Briquet infers from his own observations, more from deficient than from superabundant or too nutritious food. The entire force of this conclusion ought to be somewhat abated by the poverty of the class of patients—inmates of a hospital—with whom he has had to deal.

The various *passions and emotions*, it will be readily conceded, exert no little power over the nervous system in the production of hysteria. M. Briquet draws his conclusions on this point from observations on 430 cases. The concluding paragraph of this "article" shows the phrenological bias of the author. He adopts the philosophical divisions of mind without making use of the nomenclature of this school. It is worthy of remark, that while phrenology as a system engages public attention less now than it did some years back, its chief tenet, the plurality of the organs of the brain and corresponding plurality of the faculties of the mind, finds place frequently in investigations of a metaphysical and ethical character, and also on the subjects of education, criticism, and taste, as well as in pathology. We had occasion, in a previous number of this journal, to point out the free adoption of some of the doctrines of phrenology, in our review of Drs. Tuke and Bucknill's valuable treatise on insanity. But to the passage in the volume before us:—

"These various moral agents, by their continued action, influence disagreeably the portion of the encephalon appropriated to the affective impressions; they quicken the sensibility to such a degree that the least cause of painful sensation acting on this portion of the encephalon provokes immediately from it a reaction—that is to say, a manifestation of the uneasiness experienced—and this manifestation is hysterical."

Respecting the share which *particular callings* have on the production of hysteria, it may be readily understood that those of a sedentary kind, which keep the body in a state of prolonged rest, those which require constant stay in workshops, and those in fine, which yield insufficient salary or wages, are so many predisposing causes of the disease; while, on the contrary, those callings which demand muscular movement and which are exercised in the open air, and allow of those who work at them obtaining suitable nourishment, are so many means of warding off hysteria. This disease is represented by eminent physicians of Lausanne to be quite common in the Bernese Oberland, and some other elevated districts in Switzerland; and they attribute the cause of this frequency to the reading of romances, to the forced rest and sedentary life to which the inhabitants are subjected during six months of the year by the imbedded snow which prevents them from leaving their houses. Dr. Magnus Huss, of Stockholm, writes to M. Briquet, that the occurrence of hysteria is becoming much more frequent in Sweden among women in the country since they have ceased to work in the fields with their husbands. We can readily believe that the women of America would prefer running their chances of being hysterical to procuring exemption from the disease after the Swedish practice.

Addiction to letters and the fine arts is sometimes cited among the causes of hysteria; but, as the author thinks, without sufficient reason. Classing under the head of *professions* the members of religious orders who take the vow of celibacy, he shows that but a small number of these persons suffer from hysteria, and even of them only a few on account of giving themselves up to a life of continued prayer, seclusion, and austerities, which produce a morbid susceptibility of the nervous system. Hysteria is seldom seen among the sisters of charity who live in the open air, take much exercise, and are actively occupied.

*Servants.*—M. Briquet directs attention to women of this class in connection with the present subject, not because they are more liable to hysteria by the mere living out at service, but because their state will serve as a transition from the celibacy of convent life to the opposite habits of another class, of which notice will soon be taken. Most of these women come from the country to Paris, and suffer in consequence from nostalgia, the wearisomeness of servitude, or unusual labor, the anxieties incident to a precarious subsistence, and those growing out of their illicit unions, which lead many of them to the venereal hospital. The frequency of hysteria in this class is derivable from the causes just stated; and cannot be attributed either to unsatisfied desires or to their immoderate gratification.

*Prostitutes.*—Similiar inquiries made into the relative frequency of hysteria in prostitutes resulted in the conclusion that more than one-half of this class of females are subject to the disease; and that the proportion is more considerable in those who keep to their own rooms than the others who congregate in bawdy houses, and tramp the streets at all hours of the day and night. These latter have, in general, a strong constitution, and are careless of the future.

The causes of hysteria in this wretched class are to be found, M. Briquet very correctly thinks, in the destitution, vigils, abuse of alcoholic drinks, continual fears of the enforcement of police regulations, bad treatment on the part of the men with whom they live or consort, forced seclusion occasioned by the diseases which they contract, and unrestrained jealousy and other violent passions by which they are agitated. The frequency of hysterical attacks would probably be greater, were it not for the strength of constitution and the moral obtuseness of these women, created by their habits. Little stress can be laid on excessive excitement of the sexual organs in them, as they cease to feel venereal orgasm after the frequent performance of coition.

The next, or "Article XII.," is taken up with an inquiry into the "*influence of continence*," in giving rise to hysteria. "From the most remote times, both philosophy and medicine have regarded continence as the principal, and, indeed, the sole cause of hysteria." Plato, in whose works we find the first notice of this doctrine, says "that the womb of women is an animal which will by all means conceive, and which enters into a rage if it does not conceive;" but he gives no proof of this assertion.

In a subsequent part of the volume the author shows that diseases of the uterus and its appendages to which prostitutes are liable, have very little effect in the production of hysteria.

In recapitulating the somewhat extended arguments and details on the alleged etiological importance of continence, M. Briquet says:—

"1. That widows are not more liable to hysteria than other women.

"2. That, contrary to the assertions of authors, hysteria is of rare occurrence in women after the thirtieth year of their life, and very common before this age.

"3. That hysteria shows itself in a fifth of its cases before the age of puberty, and that then it can have no relation to the question of continence.

"4. That this disease is less frequent in married women than in single ones, only in the proportion of 7 to 9.

"5. That it is not more common in persons who live in a state of celibacy than in others in an opposite state; but, on the contrary, it may be of very frequent occurrence in those who are not at all continent.

"6. That gratification of the sensual wants gives no protection against hysteria.

"7. That it is not true that hysteric attacks terminate frequently by the evacuation of a fluid from the genital organs.

"8. That it is not true that so soon as the genital organs have reached their entire development their functions should be brought into activity, under the penalty of hysteria by the omission to do so.

"9. That it is possible, under certain circumstances of a limited range, that the genital organs, being either naturally or artificially excited and not being adequately gratified, may be a cause of strained exercise of the brain, from which cause there may arise a predisposition to hysteria; but that up to the present time this predisposition can only be received as the result of induction rather than the direct result of observation."

On the question of the influence of *menstruation* in giving rise to hysteria, M. Briquet makes the following summary, deduced from divers facts and figures in the preceding part of the article.

"1. Menstruation, whether normal or pathological, can be considered as a predisposing cause only in three-eighths of the cases of hysteria.

"2. In five-eighths of the subjects of the disease, menstruation exerted no influence in the production of hysteria, for in 87 of them the disease had appeared before the age of puberty; in 156 there was no difficulty in menstruation; and in 6 hysteria had appeared before the menopausis, (critical period.) Hysteria cannot, therefore, be regarded as the result of menstrual derangements.

"3. The effects of menstruation are felt more strongly in the period between twelve and twenty years of age than at any other time.

"4. The menstrual troubles predispose much more to the slow and gradual approaches of hysteria than to that variety in which the disease is suddenly introduced by an attack, in the proportion of 4 to 1.

"5. This kind of predisposition is only referable to the influence of the uterus and its annexed organs in a small number of cases of disordered menstruation; the greater number depending on general changes in the economy and the sufferings of other organs."

The author opens the question involved in the next article, viz., the *influence of divers morbid states* in the etiology of hysteria, by the remark that they are more influential in this way than is generally supposed. Beginning with disorders of the reproductive organs, he writes: "All that can be said up to the present time is, that of the thousands of hysterical women whose cases have been noticed by physicians, in not more than a hundred have lesions of the ovaries been met with." Nor does he believe that the predisposition to hysteria developed by uterine affections is better demonstrated than are the lesions of the ovaries in the same sense. He returns to this subject in greater detail in the next chapter, on the determining or exciting cause of hysteria. At the same time he admits that an excitable condition, in which the brain is liable to be powerfully impressed by prolonged irritations of the genital organs, may give rise, in its turn, to nervous reactions; and that the general weakness thus induced increases still further the susceptibility to morbid impressions.

*Chlorosis* shows the extent to which defective hematosis will increase nervous susceptibility, in the fact that, of 430 cases of hysteria, 152 had been very decidedly chlorotic before the appearance of the disease. *Typhoid fevers* must be acknowledged to give rise occasionally to hysteria. *Diseases of great gravity,* such as small-pox of an intense grade, pneumonia and acute rheumatism, which required an active antiphlogistic treatment, and prolonged attacks of which had called for numerous evacuations, and old intermittent fevers, caused hysteria in twenty cases of the list of 430 in M. Briquet's possession: they were developed during the period of convalescence. Some of these diseases acted as predisposing causes; others as predisposing and exciting at the same time.

There are other morbid states which seem to exert an effect the very opposite of a predisposing cause: these are organic diseases of the heart, phthisis, organic affections of the liver, and also of the kidneys, which are very rarely followed by hysterical symptoms. Even in severe diseases of the genital organs themselves, as in puerperal metritis, phlegmonous ovaritis, also in extensive pelvic abscesses—and we may add, in the great pains of coxalgia, ulcerations of the articular cartilages, and white swellings, there is still greater exemption from hysteria. This disease is sometimes caused by minor operations, such as exploration with the speculum, cauterization of a chancre, Faradisation of the skin; but never by great surgical operations.

As relates to the *state of health before the invasion of hysteria,* this, contrary to what we might suppose *a priori,* does not exert a decided influence in bringing on hysteria, or when manifested, it is in a different sense from what would have been anticipated, as we learn from the following summing up:—

"1. There is a greater readiness to be taken with a sudden attack of hysteria by subjects whose constitutions are sound than by those whose health has been previously affected.

"2. On the contrary, that hysteria which is slowly developed is more likely to appear in persons whose constitution has been weakened."

We may, says M. Briquet, sum up this long chapter on predisposition by the following conclusions:—

"1. The increased susceptibility of the affective element of the nervous system constitutes the foundation of the predisposition to hysteria.

"2. Hysteria is almost peculiar to the female sex, because there exists in it a predominance of the affective element.

"3. Nevertheless hysteria may exist in man, but on the condition that he shall manifest a similar predominance.

"4. The cause of this specialty is not to be found in the genital apparatus of a female, but in the mode of sensibility peculiar to women.

"5. Hysteria is manifestly hereditary; one-fourth of the women who are born of hysterical mothers are affected in their turn with hysteria.

"6. There exists from infancy a special state of susceptibility which is peculiar to the persons who are afterward destined to be hysterical.

"7. Hysteria may show itself in early life, before the age of puberty; the epoch of its maximum frequency is from the age of twelve to twenty-five years; beyond this latter the attacks of hysteria are of rare occurrence.

"8. We find in the development of nervous acts, more than in that of the genital organs, the causes of the tendency to hysteria.

"9. There is nothing in the temperament or physical character of the constitution which predisposes to hysteria.

"10. Hysteria is more common in the lower classes of society, in which it attacks a fourth of the women, than in the higher classes, among which it attacks an eighth of their number.

"11. Hysteria is almost as common in the country as in the city.

"12. A severe education is more apt to lead to hysteria than an indulgent one.

"13. A regimen in which the supply of aliment is insufficient more readily tends to the production of hysteria than a too nutritious diet.

"14. The passions and moral affections of a sad nature are the only ones which predispose to hysteria.

"15. The professions (and callings) only have an influence in predisposing to hysteria, in so far as they may weaken the constitution, by giving a predominance to the nervous system, and increasing the occasions for painful impressions on this system.

"16. Diseases of the genital organs do not predispose much more to the supervention of hysteria than the diseases of other organs.

"17. Continence predisposes to hysteria in a very small number of cases, in which, from peculiar circumstances, the genital organs are excited, or in which the portion of the brain that corresponds with the correlation of the actions of these organs is similarly affected.

"18. There is nothing in its predisposition which justifies the opinions of the ancients on the physical causes of hysteria."

*Determining or exciting causes of hysteria.*—M. Briquet, in the chapter devoted to the subject, enumerates those causes which have been supposed by different writers to be efficient in the bringing on an attack of hysteria, and comments on the fallacies of opinion exhibited in this catalogue. He then specifies the causes which he believes to be those that really deserve the name of exciting ones. The deductions are made from 594 hysterical subjects. Omitting the first-mentioned enumeration of authors and his notices of certain cases, we extract his closing summary, viz. :—

"1. In five-sixths of hysterical cases, the disease has followed the action of an exciting cause.

"2. In a seventh of the cases, it was developed under the sole influence of predisposition.

"3. In the proportion of a fiftieth, it was produced without any appreciable cause.

"4. More than half the causes acted directly on the brain. Three-fifths of these operated by debilitating the organism and producing a predominance of the nervous system. The sixth displayed its primary action on the stomach. The eighth had, at first, acted on the uterus and its appendages; finally, causes the proportion of which it is difficult to appreciate, act on the external tegument, but all eventually acted on the brain.

"5. The importance attached to the influence of the genital organs has been excessively exaggerated.

"6. The causes producing hysteria, when ushered in by an 'attack,' are commonly instantaneous and powerful.

"7. Those which gradually produce hysteria are, on the contrary, slow, of little intensity, and of such a nature as to alter the constitution.

"8. The causes of hysteria, studied in reference to age, show the little influence exerted by the genital organs and the great power of the different parts of the brain."

Contrary to what might be supposed *a priori*, the difference between the causes of hysteria in the poor and in the rich is not great.

The *second part*, constituting more than a third of the volume, is devoted to the "symptomatology" of hysteria. It opens with chapter third, and its first article is on the "prodromes" of the disease. M. Briquet relates, at the outset, the history of a case, in which is exhibited a development of the various disturbances that go to make up hysteria, during a period extending from infancy to twenty-five years of age. Contrasted with this is the history of another case, in which hysteria manifested itself at a late period in consequence of occasional causes.

The premonitory symptoms of hysteria are displayed in three different modes.

"1. It begins at a very early age, either through hereditary transmission, or through a special constitution, or, finally, under the operation of long-continued and painful sensations. This mode of beginning hysteria prevails in a sixth of the entire number of cases. The children in whom the disease thus shows itself are very excitable, morose, and irritable, and disturbed by the slightest contradictions; they become pale or flushed, complain of a sense of suffocation, sob, are seized with a choking, palpitation, agitation, and trembling of the limbs; sometimes syncope supervenes. They are sufferers before long from headache, disturbed sleep, and painful dreams; they awake in a fright or are attacked with nightmare, and sometimes with somnambulism. The epigastrium is the seat of pain, the appetite is variable and capricious, and the digestion painful; sometimes there is vomiting; finally, pains are felt in the back and between the shoulders, and on the left side of the chest.

"2. At other times, and in rather less than half the number of cases, hysteria is developed at a more advanced age, and in a gradual manner, under the following circumstances. Sometimes the subjects are young girls, of medium strength, in whom hysteria is produced by fatigue and bad food, or ennui; the menses are then irregular, and are finally suppressed. At other times we meet with married women who are loaded with cares, or tormented either by bickerings or by other vexations of housekeeping. In both cases the patients become pallid and thin, their susceptibility is extreme, their temper is soured; they are put out by the slightest cause; under the least emotion there supervene tears and sighs, a sense of choking, oppression at the epigastrium, agitation of the limbs; then cephalalgia, palpitations, hurried breathing, and all the phenomena of chlorosis: the constitution is changed, the nervous system assumes the preponderance, and the digestive functions are deranged. Gastralgia with its whole train of anomalous sensations appears; pains in the back and on the left side of the chest show themselves; restlessness and formication are felt in the limbs."

To these prodromes may be added, in not a few cases, great fickleness of disposition, odd ideas, painful thoughts, tears and laughing without motive, sleeplessness or disturbed dreams; sometimes wandering chills, sometimes a burning heat, and again, frequently, an icy coldness of the hands.

3. In fine, in a certain number of cases which may be estimated as a third of the whole, there is no precursory phenomenon of an appreciable nature; and the female appears to be in a state of full health, when, on the occasion of a great fright or a strong moral emotion, there supervenes, either at the moment of the occurrence of the cause or some hours afterward, an attack of hysteria with convulsions and entire loss of consciousness.

Who can fail to see, says M. Briquet, in these prodromic disorders, which constitute what some writers have called hystericism, an evident cerebral disturbance, with all the signs of congestion of the brain; then an influence radiating from this nervous centre to the different divisions, splanchnic, muscular or sensorial, of the nervous system (reflex action)? It would be hard to find any primitive action of the genital organs under these circumstances.

Under the head of *symptoms* proper, the list is tolerably long. Some of these, as remarked by the author, are of unfailing occurrence, such as extreme excitability, pains in the epigastrium, on the left side of the chest and along the sulcus on the left side of the vertebræ. Other symptoms are less constant, but are generally met with, and constitute the foundation of the disease; such as various hyperæsthesia, convulsive attacks, paralysis, etc. There are others again which are of occasional occurrence, and are only produced under the influence of particular conditions in which hysterical subjects find themselves. To this class belong delirium, lethargy, catalepsy, ecstasis, somnambulism, etc. These various phenomena may, M. Briquet thinks, be divided into eight classes, viz.: 1. Hyperæsthesia. 2. Anæsthesia. 3. Perversions of sensibility. 4. Spasms. 5. Attacks of spasm, convulsions, catalepsy, somnambulism, ecstasis, coma, lethargy, and syncope. 6. Paralysis. 7. Perversions of contractility. 8. Modifications of exhalation and secretion.

*Hysterical hyperæsthesia* is distinguished from other varieties of morbid sensibility by its being seated in certain organs preferably to others: it is more changeable in its seat than the other kinds of hyperæsthesia, and is especially influenced by moral affections. It may appear in all the organs to which the nerves of relation are distributed; and in a particular manner the skin, the muscles, the articulations, the nerves, the organs of the senses, the larynx, the bronchiæ, the intestines, the kidneys, the bladder, and the uterus. Hysterical *dermatalgia* or hyperæsthesia of the skin, although it must always have occurred, was only noticed for the first time in a formal manner by MM. Moneret and Fleury, in 1842. Cases have occurred in which there was such exquisite sensibility of the left mamma as to induce surgeons, who were considered skillful, to recommend excision of the organ. Dermatalgia is seldom met with except in acute cases of hysteria.

*Myosalgia* or muscular pain is of such common occurrence in hysteria, that there is not, the author assures us, a single case of an hysterical female who has not suffered from it in the course of the disease. Most of the troubles in hysteria are connected with, or end in irregular actions of the muscles, as we see in convulsions, paralysis, anæsthesia, and most of the muscular pains. Exponents and ready servitors, as the muscles are, of the various emotions and passions, it is easy for us to understand why they should be called into violent and irregular action in hysterical attacks, in which the sufferer is so often a prey to these states of mental disorder. The muscular pain is simply a perversion of sensibility, and is in no way associated with inflammation.

Following out the varieties of hysterical myosalgia, M. Briquet speaks in succession of *cephalalgia, epigastralgia, rachialgia, pleuralgia, costialgia, thoracalgia, myelosalgia, arthralgia, neuralgia, hyperæsthesia of the organs of the senses, laryngo-bronchial hyperæsthesia, hysteric cough, pseudocroupal suffocations, asthma, gastralgia, enteralgia, nephralgia, cystalgia, hysteralgia.* Many an instructive lesson is given in the observations and deductions made by the author under these several heads. That which ought most strongly to impress the mind of the practitioner is the extreme importance of a correct diagnosis in affections of the different organs, so that they may not be supposed to be the seat of inflammation, when, in reality, their muscular tissue is suffering under a morbid excitement of the nerves of the part, or from neuralgia and myosalgia.

In the headache of hysterical subjects, the pain is chiefly confined to the fleshy portions of the muscles of the cranium. The *clavus hystericus*, a rare variety of cephalalgia, has most commonly its seat in the temporal regions and in the sinciput; it is confined to one spot, which is very circumscribed: the pain is intense, and may last for several days. *Epigastralgia*, like cephalalgia, attacks the largest number of hysterical subjects, and ushers in the paroxysm. Sometimes it results from direct irritation transmitted from the nervous centres; sometimes from reflex action, beginning at the digestive canal, which was the seat of disorder. It interferes with the movements of the respiratory muscles, and when the diaphragm is prevented from its free play, the distress is great. The least ligature, even the pressure of ordinary clothing, cannot be borne. *Rachialgia*, by which the author means, hyperæsthesia of the muscles of the back, viz., the trapezius, the dorsalis major, and the mass consisting of the sacro-lumbalis, and longissimus dorsi, is also one of nearly constant occurrence in hysteria. It affects the upper half of the spinal region in preference to the lower; and the left rather than the right side, and in point of frequency comes after cephalalgia and epigastralgia.

M. Briquet quotes Sir Benjamin Brodie, on the egregious blunder in diagnosis, by which rachialgia is confounded with a disease of the spinal marrow, and a young female is condemned, in consequence, for many years, to a recumbent posture, and to be tormented with setons, moxas, and cauterization, when in fact fresh air, exercise, and suitable amusements would have effected a cure in a few months. The French author corroborates this view, and tells us that the mistake is quite a common one.

*Pleuralgia* commonly extends over a portion of one side of the chest, sometimes in an oblique direction, corresponding with that of the ribs, sometimes directly horizontal, as a belt, equal to four or five fingers' breadth, on a level with the fifth, sixth, seventh, and eighth ribs and the intervening intercostal spaces, giving a preference to the left side. Pleuralgia, intermediate as its seat is between rachialgia behind and epigastralgia in front, has been confounded with intercostal neuralgia, of which the first-mentioned, hyperæsthesia, was represented to be the vertebral end, and the second the abdominal; all three being supposed to depend on a lesion of the intercostal nerve.

*Cælialgia*, or hyperæsthesia of the muscles, anterior and posterior, which compose the walls of the abdomen, offers some interesting points of diagnosis; as when pain on either side of the hypogastric region, evinced on pressure, is supposed to indicate a phlogosis of the ovaries. The error is rectified by making the slightest pressure, or even slightly rubbing the surface, so that the muscles alone are compressed, and not the subjacent parts. So, also, pressure in the hypogastric region, when productive of great pain, is assumed to evince uterine inflammation, when in fact it is often only a proof of hyperæsthesia of the lower part of the recti and the pyramidal muscles. Thoracalgia, or hyperæsthesia of the anterior muscles of the thorax, is not common in hysteria. *Myelosalgia*, or pain of the muscles of the limbs, has been noticed by M. Briquet in sixty-four cases of hysteria. Sometimes it is combined with dermatalgia, and often with anæsthesia of the corresponding portion of the skin.

*Arthralgia*, as a hysteric affection, was first clearly noticed by Frederick Hoffman; but the merit of successful generalization, and of fixing on it the attention of the profession, belongs to Brodie. Commonly the pained joint exhibits neither redness, nor any appreciable heat of the skin, nor swelling, nor any sign of hydrarthosis in the synovia of the diseased joint. Neuralgia, contrary to the opinion of most writers, is, we are told by M. Briquet, a rare affection in hysteria, and of which, when it does occur, it must be considered a complication, rather than an essential part.

*Hyperæsthesia of the organs of the senses* gives, at times, in hysteria, an astonishing acuteness to the functions of these parts. To this circumstance we must refer the alleged marvels, told some years ago, of young girls who could read with their eyes closed, and who in fact had hyperæsthesia of the optic nerve, which allowed of their reading with almost a meeting of the eyelids.

*Hysteric cough* has been so well described by Sydenham as to leave little room for improvement by his successors. We should like to note some of the points laid down by M. Briquet on this occasion, but have not room for the purpose. Looking to the frequent fits of coughing, twelve to fifteen in a minute, for hours entire, it is not easy to divest one's self of anxiety for the result, which experience shows, however, to be favorable. *Pseudo-croupal suffocation*, an alarming feature, and of sudden occurrence in hysteria, may be promptly and completely relieved by tracheotomy, and it is in such cases, much more than in ordinary croup, that there ought to be no hesitation in having recourse to the operation. Asthma is now and then met with as a hysteric complication.

*Gastralgia and enteralgia* constitute *hyperæsthesia of the digestive passages.* Gastric disorder is the most frequent prelude to hysteria in those cases in which the disease comes on gradually. Capricious appetites and aversions to certain articles of food are common in hysteria. In some, vomiting is the predominant symptom, associated with gastralgia; but both affections yield to remedies and time. The case is different when the pain is acute and violent and the stomach rejects all food. Prompt and efficient measures are then called for. Moral affections readily induce hysteric gastralgia. Sometimes it disappears suddenly. *Nephralgia* and *cystalgia* should be thought of in probable connection with hysteria. Hysteralgia, though a painful disease, seldom attacks hysterical subjects.

*Anæsthesia*, the opposite state to that which we have just been considering, is described by our author, as regards the first notices of its detection and its now recognized features and peculiarities, with more fullness than we can give an idea of in our very curt analysis. Our waning space compels us to increased compression, even although it be of rich and varied material. In times gone by, the fact of persons having certain parts of the body insensible to ordinary impressions and to painful irritations subjected them to the imputation of sorcery and dealings with the devil, and brought on them the punishment of death. Anæsthesia is so common in hysterical subjects, that some writers believe in its being always present in one part or another of the organism of such persons. It frequently supervenes on attacks of hysteria. Its seat is always in the organs or tissues supplied by the cerebro-spinal nerves, and never in those of organic life: its duration varies from days to years. The skin is the most common seat of anæsthesia, and next to it the mucous membranes at the openings of the different cavities which they line. Anæsthesia in the eye may, in the last degree, give rise to amaurosis; in the ear it seldom goes to deafness. Muscular anæsthesia has given rise to some strange scenes, as in the stripes, blows, and self-imposed tortures to which fanatical subjects, in a state of hysteria, were subjected, without their evincing any sensibility, or even an approach to pain.

A short chapter is devoted to the subject of "*perversions of sensibility*," in which many curious cases are related. Among these was that of Weber, the celebrated musical composer, who fell into syncope from the odor of Cologne water. A more noticeable fact, as serving to explain some of the mummeries of spirit-rapping, is the sharp noise similar to that made by the joints of the fingers when they are suddenly and strongly stretched. This noise is heard at the time of the disappearance of hysterical convulsions or cramps. M. Briquet relates an instance of this cracking sound, which could be produced at will by a woman moving her scapula, while raising her shoulder, and which was owing to the contact of the anterior face of the scapula with the posterior parietes of the thorax.

"*Spasms*" are the subject of chapter sixth of the volume before us. They have been defined to be, convulsions of the muscles of nutritive life and of the elastic tissues; that is to say, of the contractile apparatus which are not under the direct control of the will, and which receive their nerves, in great part, from the trisplanchnic, and in small proportion from the cerebro-spinal system. These spasms may affect the organs of digestion, respiration, and circulation, and also those of the genito-urinary apparatus.

*Spasms of the pharynx and œsophagus* give rise to the sensations of choking and strangulation, which are such common symptoms of hysteria. The spasm of the throat, or hysterical suffocation, may have its seat in the pharynx and œsophagus, or in the larynx and upper portion of the trachea. For the most part it only comes on during an attack of hysteria, or from strong mental emotion. The pharyngo-œsophageal constriction sometimes lasts but a few minutes, and ceases with the attack of hysteria; but it may continue for several days, the patient during this time being unable to eat or drink. In one case, the patient, in a period of seven months, was unable to swallow anything except a little broth. Vomiting is one of the effects of a spasmodic condition of the muscular coat of the stomach. It may continue for months; for, like hiccough, it is a very persistent symptom. In some there is so complete a peristaltic action that even enemata have been thrown up by vomiting. Borborygmi are common enough in hysterical subjects; at times the gas in the intestines is confined between two points of contraction of the muscular coat, and there result pains and globular tumors, which may occupy successively, and in a short period, all the different parts of the abdomen.

*Suffocation, barking, mewing, etc.*—When the spasm attacks the air-passages, the muscles of the larynx are thrown into a convulsive state, and there succeeds a series of diverse phenomena. When it is confined to the constrictor muscles of the glottis, which receive branches from the pneumo-gastric and the two upper cervical ganglions, the breathing is impeded, and the inspiration labored and hissing. When it extends to all the muscles of the larynx, the voice is suddenly lost, or it is greatly changed, becomes acute, and constitutes a kind of cry. When the spasm seizes, at one and the same time, the muscles of the larynx and those of the chest, of the abdominal parietes, and of the diaphragm, there ensue those varieties of sounds which have excited the astonishment of observers. These cries, says Willis, imitate the barking and howling and yelping of dogs, the mewing of cats, the bellowing of cattle, the chucking of fowls, the grunting of hogs, and the croaking of frogs.

These cries are of variable duration, and recur at variable intervals. Sometimes they are intermittent, as in the instance of the nuns of a convent, who were quiet during the day, but when six o'clock came, began mewing, and continued it the whole evening. In some cases, the patients, instead of uttering cries, give way to vociferations in the same tone, or repeat continually the same word or a very short phrase. One case related by the author was cured by moral means, which consisted mainly in taking the patient abroad in the most public streets of Paris, and acting on her sense of shame at exposing herself to the public gaze and the comments of those she met, if she yielded to the utterance of strange cries or other noises. There are not wanting cases of hysterical seizure in which the patient, a female, highly educated, refined, and delicate in her deportment and language when in her ordinary state of health, would indulge in coarse and vulgar speech, and even obscene epithets.

M. Briquet refers to the histories of epidemic barking, given by different authors. In boarding schools and in nunneries this disorder has been known to spread rapidly from a single case, in virtue of imitation by those around, whose susceptibilities readily predisposed them to nervous disorder. *Hiccough*, a common occurrence in hysteria, may persist all day, but invariably disappears during sleep.

*Spasms of the organs of circulation*, as evinced in palpitations, with a very frequent pulse, sometimes one hundred and thirty to one hundred and eighty in a minute, may come and go suddenly, or may last during months and even years; the skin all the time preserving its natural temperature. Spasms of the constrictor muscle of the vagina have been noticed in some instances, and in others of the neck of the bladder, accompanied with retention of urine.

Chapter seventh is devoted to a consideration of *hysteric attacks or paroxysms*, which, from their marked character and sudden cessation and recurrence at intervals, were supposed by some writers, as Houillier and Riverius, to be remittent. It is more correct to believe in the continued character of hysteria itself, and its occasional aggravation by the occurrence of paroxysms. These may present themselves under different forms, which are reducible to the following, viz., 1, spasms; 2, syncopes; 3, hysterical convulsions; 4, epileptic convulsions; 5, catalepsy; 6, ecstasis; 7, somnambulism; 8, coma and lethargy; 9, delirium. Under these several heads the author introduces a large amount of description of curious morbid phenomena, historical incidents, and details of cases connected with the occurrence of hysteria. The article on hysterical convulsions is particularly full, were it only in the accounts of different epidemics of the disease during the sixteenth and seventeenth and in the early part of the eighteenth century. The period of life in which these convulsive attacks are of most frequent occurrence is between fifteen and thirty years of age. A singular case of a young girl, twelve years of age, is related, which we wish much that we had room to introduce here, but cannot. One of her extraordinary feats in the hysteric paroxysm was, to be able to dance, not only on a table, but on a stick placed transversely, also on the back of a chair, and on the shoulders and on the head of another person, without losing her balance. She heard nothing, and was insensible to pain and punctures, made with a view of causing pain. The tendency to imitation is very strong among females disposed to hysteria, and hence the succession of attacks in one person after another, where the first sufferer is seen by her companions or friends. Many of our readers may remember the manner in which the celebrated Boerhaave arrested this imitative tendency among young persons of both sexes to fall into epileptic convulsions, in the hospital at Haarlem. He assembled all the inmates of the hospital, and directed iron rods to be heated in their presence, after which he declared in a loud voice that, since all other means had been found of no avail, he knew of but one remedy to be employed, which was to burn with a red-hot iron a particular part of the arm, down to the bone, of the first person who might be taken with convulsions. The scene, the loud voice and impressive manner of Boerhaave, impressed so strongly the children of the hospital with a fear of the operation as to enable them to resist the approaches of the next paroxysm.

In speaking of the diagnosis of hysterical convulsions, M. Briquet indicates the characteristic symptoms of hysteria and of epilepsy in parallel columns. The distinction between the former of these two diseases and eclampsia, and lead colic, and convulsions, is also pointed out. A section or "article" is given to a notice of epileptic attacks which may be combined with hysteric ones. The union was called by Sennert *epilepsia uterina*, a name indicative of the dominant etiological creed of the time, and which M. Briquet has taken so much pains to nullify.

We must pass over, without special notice, what we find in the sections on attacks of catalepsy, ecstasis, somnambulism, sleep, coma, lethargy, and delirium, also of convulsions and rigidity of the muscles, in the interval between the attacks of hysteria. Paralysis in different degrees is quite common in hysteria; it is only seen in the muscles supplied by the cerebro-spinal nerves; the hemiplegic is the most common variety, and this on the left side. The "article" on this theme is full and instructive, and is further enriched with details of cases.

The *third part* of the volume opens with chapter thirteenth, on "the march [or progress] of hysteria," which, unlike that of so many other diseases, does not go through a series of successive periods. The author treats of it under the divisions of acute hysteria, hysteric fever, slow hysteria, and hysteria with intermissions. To these succeed causes of intermissions, and sudden cures. The next chapter treats of the "complications" of hysteria, and the following one of its "terminations." After this we meet with the chapters on "the pathological anatomy of hysteria," its "diagnosis" and "prognosis," and "the seat and nature of hysteria." Morbid anatomy has failed to reveal anything positive respecting the seat or the nature of hysteria, for all the lesions that have been noticed in the *post-mortem* examinations of hysterical subjects are the results of complications of disease in other organs, in addition to those of the nervous system, which is the real seat of hysteria. Rarely do females die from this disease—divested of the complications just mentioned. We the less regret our not being able to repeat what the author has said on the subject of diagnosis, as nearly all of it has been anticipated in the description of symptoms, to which we refer our readers. The hysteric tripod, according to the author, consists of the three hyperæsthesiæ, viz., 1, epigastralgia; 2, a pain on a level with the middle portion of the left false ribs, or pleuralgia; 3, rachialgia. If to these we add the frequent presence of anæsthesia, chronic convulsions, and paralysis on the left side of the body, we shall have little difficulty in making out a correct diagnosis of hysteria. The prognosis of hysteria, by many writers, is quite favorable; but when we reflect on the morbid sensibility which it leaves behind, the great exhaustion following its repeated attacks, and the extreme difficulty of curing it, as the conditions for doing so are not under medical control, we cannot regard it otherwise than as a very formidable disease.

In discussing the questions of the seat and nature of hysteria, which M. Briquet does at some length, he repeats some of the arguments and views which he had advanced when he spoke of the predisposing causes of the disease. After passing in review the different pathological creeds on these points, be reaches the conclusion, that hysteria is a passionate manifestation of the suffering or irritation of that portion of the encephalo-spinal mass destined to receive the affective impressions and sensations. That view which would place the seat of hysteria in the spinal marrow is entirely too restricted, and excludes an admission of the agency of moral causes, which are so powerful, and which for their manifestations require the medium of the brain.

*Treatment.*—The *fourth* and concluding *part* of M. Briquet's work is on the treatment of hysteria. It is divided into three chapters, viz., on prophylaxis, the treatment of hysteria proper, and the special treatment of each of the symptoms. The prophylactic treatment ought to begin with a hygienic course, to be pursued during the pregnancy of the mother, under the supposition that the coming child may be a female. The indications, in this case, will be to diminish as much as possible the excessive excitability of the nervous system, to augment the activity of the muscles, and to furnish to the blood the elements which shall place it in a normal condition. With this view, the future mother will retire to the country, take regular and moderate exercise, make for herself pleasant occupations, and avoid indulgence in strong emotions and passionate excitement. An hysterical woman ought not, M. Briquet thinks, to nurse her child: his belief is, that she will not furnish it with all the elements or with those of a good quality for proper hematosis, and it is not certain that her milk may not contain elements which will augment the nervous susceptibility of the child.

In confiding the little being to a nurse, care must be taken that this person is in good health, is of a sanguine constitution, and calm in her disposition, and not excitable. Aware of the hereditary character of hysteria in one-fourth of the cases of the disease, we should advise that the education of the child must begin at its birth. Accordingly, says our author, the indispensable conditions for the education of the first period of infancy are, a good milk diet, residence in the country, habitual exposure to the outer air, and reserve in the expressions of tenderness on the part of the mother and of the relatives in habitual intercourse with the child. So soon as the latter can walk, it ought to be clad in easy-fitting garments, and of a texture as light as is compatible with the state of the weather. In fine, all the rules of sound hygiene, both in its physical and moral relations, should be carried out, with a preference to what in the language of the day is called "roughing it." The time should be divided between intellectual and bodily exercise, an arrangement very different from, but far preferable to, that faulty system now in vogue, in which scarcely an hour in the day is left open for exercise and recreation.

At the epoch of puberty, and when the young female is about entering into the world, to act her part in it, increased vigilance should be exercised to enlist all the means—hygienic, moral, and intellectual—which may give vigor to her frame and stability to her mind, especially by her avoiding whatever may increase excitability or favor false and exaggerated sentiment. Crowded assemblies, balls, and the theatre should be avoided. The reading of novels, by giving erroneous and fanciful views of human nature and actions, and indisposing to a discharge of the practical duties of life, ought also to come within the prohibited list. Tissot has said: "if your daughter reads romances when she is fifteen years of age, she will have the vapours at twenty." In their intercourse with young women predisposed to hysteria, the parents ought to appeal to reason more than to sentiment, and show them that their extreme sensibility subjects them to ridicule and places them in the power of those around them. The present practice of treating children with extreme care and tenderness and nurturing their sensibilities, is, M. Briquet thinks, less favorable to a healthy state of the feelings, than the more precise, formal, and exacting manner of parents of a former generation.

Among the means preventive of hysteria, and also those of cure when it has appeared, marriage has been recommended on the high authority of a long succession of writers. The question is examined by M. Briquet under various aspects, and with evidence of considerable literary research, which we are the less obliged to repeat here, after what was said on this subject in treating of the predisposing causes of hysteria. Physical as well as physiological considerations must enter into a solution of the question. A girl, neglected, lonely, and discontented under the parental roof, may find in the husband of her choice a new life opened to her—greater variety in occupation and in the exercise of her affections; and in this way through a genial excitement of her nervous system nutrition becomes active, she recovers her complexion, and gains rotundity of figure. But in all this we must find more varied and multiplied causes than in the simple excitement of the genital organs and gratified sensual desires. Let the scene in married life be changed; let crosses, contentions, bad conduct of the husband and anxieties about the children disturb the mind and derange the harmony of the nervous system, and then will ensue hysteria and its associate disorders, although the sexual desires continue to be gratified and the condition of the entire reproductive apparatus have been changed. Of 421 hysterical subjects, whose cases were studied by M. Briquet, 152 were married, or had kept house and borne children, and of the others, eighty-seven were of infantile age, or under twelve years.

More reliance is to be placed, as remedial means, on an entire change in the external life of the patient, by change of scene, travel, prolonged voyages, visits to watering places, meeting new faces, and making new acquaintances. A change of constitution may also be effected by the use of analeptics, nourishing food, in which milk enters largely, chocolate of a proper manufacture, as of cacao and sugar, or with feculant articles, as in broma. The use of red, especially Bourdeaux wines, is much insisted on by the author, or if not procurable, beer in its place. Chalybeates mixed with wine, at meals, are also well spoken of; and M. Briquet advises, at other times, hydrogenate of iron, the pills of Blaud, Vallet, the lactate of iron of Gelis and Conte, and the ferruginous syrup, to be continued with some persistence. Of the vegetable tonics, a preference is given to the gray bark in powder, in doses of a few grains, also the cold infusion or the vinous syrup. Mineral waters take high rank among the prophylactic remedies, as they do also for the cure of the disease, when it has come on. The same may be said of the cool bath, from sixty-five to seventy-five degrees of Fahrenheit, followed by frictions with flannel dipped in some aromatic spirit.

There are, on the other hand, females of a sanguine temperament and a full habit who are predisposed to the disease and become hysterical. They require the use of diluents and demulcents; restriction to white meats, whey, etc., and prolonged immersion in a cool bath of the temperature just indicated.

The *general treatment* of hysteria is carried out by the use of the following classes of therapeutical agents, viz.: stimulants; antiphlogistics; hydrotherapeias; antispasmodics; narcotics, and revulsives. The first class, or stimulants, M. Briquet believes to be seldom called for or beneficial in hysteria. Tonics, on the other hand, are of marked utility, as might be inferred from the debility and anæmia which are so common in the subjects of this disease. Among the antiphlogistics, blood-letting is seldom serviceable, except in a few cases of plethoric habits, in which its use should be clearly indicated. We must pity such patients as the young hysterical woman, at Turin, who was bled 700 times in fourteen years; and a woman of Alexandria, who was bled 1400 times in eight years, and, the most extraordinary part of the story, who was eventually cured. Leeches to the anus or vulva have been found serviceable, however, in the hemorrhagic diathesis, and in suppression or absence of the menses, and in habitual discharges of blood. Tepid, or rather cool baths as above, so highly extolled by Pomme, should be used every second or third day, and in some cases even every day; prolonging the duration of each bath to five or six hours. Tepid or cold enemata often give great relief. Under the head of "hydrotherapeia," M. Briquet renews the expression of his confidence in the remedial efficacy of cold, and cites his success in typhoid fever by cold bathing, ice to the head, iced drinks and iced enemata. In acute hysteria, as in hysteric fever, delirium, sleeplessness, extreme restlessness, convulsions, erethism, and excessive general excitement of the organism, he extols this treatment. Contributing to the same end are hip baths and pediluvia in water of the temperature of 56° Fahrenheit. The hydropathic treatment, intermittent or continued, by a packing in wet sheets, is well spoken of: the first or intermittent, is to be had recourse to every third or fourth day: it will consist of a single packing and reaction. The other consists in the repeated application of ice to the head in cephalalgia and delirium, and to the abdomen and pelvis in hyperæsthesia of these parts.

*Antispasmodics* rank low, in the therapeutical scale of the author. Very different is the value he attaches to *narcotics*, as represented more particularly by opium and its salts, the use of which in considerable and repeated doses is tolerated to a great extent by hysterical subjects; and hence the doses may be increased until it produces the desired tranquilizing effect. The auxiliaries to opium specified by the author are, distilled water of the lettuce, the syrup of lactucarium of Aubergier, in teaspoonful doses several times a day, the distilled waters of the cherry-laurel and of the black cherry, infusions of peach flowers, the cherry-laurel, bitter almonds, bruised, which make so many varieties of pleasant drinks, and the habitual use of which ought to be recommended to hysterical subjects. This treatment, which M. Briquet assures us is the best borne by this class of patients, should be continued perseveringly for a period of two months, and will be rewarded by successful results. It is the standing treatment, but it need not interfere with the use of chalybeates or of remedies applied to the relief of particular symptoms. Magnetism, (animal,) which the author calls fascination, is a tranquilizing agent, and may be used with good effect in some cases: its operation is, he alleges, entirely due to its acting on the imagination and the senses, and not by any material agency. It ought to be regarded as an anæsthetic, and to be only occasionally employed to abate some unexpected disturbance. So, also, of chloroform; its effects are transitory and exert no permanently beneficial influence on the disease.

*Revulsive medication* is one of the most useful means of treating hysteria. The revulsives most in use act primarily on the skin. Electricity may be regarded as the most efficient of the entire class; the preferable method of using it is by giving rise to currents by induction, constituting what is now termed Faradisation. The best apparatus for the purpose is that of M. Duchenne. The benefits from Faradisation, as a means of counter-irritation or of revulsion, are to produce at will a pain and irritation in all possible varying degrees of intensity, and to be able to prolong this pain and excitation during some minutes consecutively, while leaving the skin perfectly sound, and with ability to apply it to every part of the body.

The last chapter of this volume is on "*the special treatment of each of the symptoms of hysteria.*" Compelled to draw our analysis to a close, we can barely notice some few of the leading points in the detailed directions for the administration of therapeutical agents. These are to be used in order to meet the indications furnished by the prominent forms of disorder which present themselves in hysteria. The difference between this and the preceding chapter consists not so much in any novelty of means in the latter as in their specific applications to existing exigencies. For the relief of dermatalgia, the chief treatment will consist of revulsion in the manner already mentioned. Hyperæsthesia of the muscles is met by antiphlogistics, antispasmodics, anodynes, revulsives. "The sole anodynes, the use of which is followed by real and constant effects, are applications of cold water and ice." "But the truly heroic medication of hyperæsthesia of the muscles consists in the employment of revulsives," in which Faradisation plays an important part. Hysterical rachialgia is relieved by similar means. The neuralgia of hysteria requires the same remedies as ordinary neuralgia. Hysteric cough is but little relieved by medicine: its cure is to be looked for in a general treatment consisting of tonics and hygienic agents which will give tone to the economy. Nearly the same remarks apply to the treatment of gastralgia, with, at the same time, a removal, if possible, of the causes of the disease, and especially those of a moral kind, and the substitution of pleasurable emotions, to be procured in part by travel and varied occupation and amusement. So also of enteralgia. Hysteralgia, for which so many remedies have been given with indifferent success, has been promptly relieved by an incision on the neck of the uterus in the direction of the axis of this organ, as recommended by M. Malgaigne. M. Briquet prefers currents by induction, made to pass through the most painful part of the neck of the uterus. For the removal of anæsthesia of the skin, "the most certain, the most simple, and the most expeditious, is Faradisation of the affected part." With it we may conjoin, if necessary, the revulsives named in a preceding page. The prime remedy, Faradisation, is, the author assures us, the remedy for nearly all the other varieties of anæsthesia, as it is met with in the organs of the senses and in those of nutrition. Spasm, as dysphagia, constriction of the throat, and of respiration, barking, etc., are benefited by this measure, evidently a very favorite one with the author. He recommends, also, in the last-mentioned disorder, regular exercise of the organs of voice and speech, as in declamation and singing, while beating time. The most reliable remedies for vomiting are icy-cold gaseous drinks, swallowing pounded ice, sinapisms and blisters, and finally a bladder filled with ice applied to the epigastric region. Seldom will vomiting resist these means if aided by opiate enemata. In the most obstinate cases, all drinks are to be withheld, and only some spoonfuls of pounded ice are to be taken, and the skin of the epigastric region is to be Faradised.

For combating the paroxysms or "attacks" of hysteria, M. Briquet advises different means, with, however, a special preference for electricity in the manner so repeatedly mentioned. In paralysis following hysteria, this remedy has done wonders, performed almost miracles. Cold baths, cold affusions, and cold douches, directed on the affected limbs, are also highly praised.

